# Bayesian spatial modeling of measles outbreak in Texas, USA

**DOI:** 10.1007/s10651-026-00718-5

**Published:** 2026-03-30

**Authors:** Gihani V. W. W. Disanayakage, Asim K. Dey

**Affiliations:** Department of Mathematics and Statistics, Texas Tech University, Lubbock, TX USA

**Keywords:** Bayesian inference, Measles in Texas, Spatial effect, Zero-inflated Poisson

## Abstract

Measles remains a significant public health concern despite the availability of an effective vaccine, with recent outbreaks in Texas illustrating ongoing vulnerabilities in disease control. This study investigates the spatial distribution of measles incidence during the 2025 outbreak period across Texas counties using a Bayesian Zero-Inflated Poisson model with spatial dependence, incorporating a conditional autoregressive prior. The model accounts for the excess zeros in the data, i.e., counties reporting no cases, while capturing spatial correlations and the effects of demographic and environmental covariates, including MMR vaccination rates, population density, temperature, and precipitation. By applying this modeling framework, we estimate key epidemiological metrics such as the standardized incidence ratio, relative risk, and exceedance probabilities to identify high-risk regions. Results highlight a pronounced spatial concentration of elevated measles risk in northwestern Texas, where low vaccination coverage and other contributing factors have led to a severe outbreak.

## Introduction

Measles continues to pose a serious global health risk, despite the existence of a highly effective vaccine. It causes symptoms like high fever, cough, conjunctivitis, and a telltale rash. In severe cases, especially among young children and immunocompromised people, it can lead to complications such as pneumonia, encephalitis, or death (Leung et al. 2024; Lindquist and Lindquist 2020; Centers for Disease Control and Prevention 2025). Measles is one of the most contagious viral diseases, with a basic reproduction number ranging between 12 and 18, meaning one infected person can, on average, infect 12–18 others in a susceptible population (Guerra et al. 2017; Moss 2017). Although the introduction of the measles-mumps-rubella (MMR) vaccine in the 1960s and subsequent widespread immunization campaigns led to the declaration of measles elimination in the U.S. in 2000, recent years have witnessed a troubling resurgence of cases in several regions, including the state of Texas (Patel et al. 2019; Centers for Disease Control and Prevention 2025). The recent measles outbreak in Texas has been the most severe in Gaines County, located in the South Plains region along the New Mexico border. As of March 2025, Gaines County reported hundreds of confirmed cases, accounting for a significant portion of the state’s total cases. This area has also experienced tragic outcomes, including the deaths of two children due to measles-related complications (DSHS 2025).

Understanding the drivers of measles outbreaks in Texas requires a multifaceted approach that considers both epidemiological and environmental factors. Vaccination coverage, particularly the rate of administration of the MMR vaccine, plays a critical role in determining community immunity levels. The Centers for Disease Control and Prevention (CDC) recommends a two-dose regimen of the MMR vaccine, which is approximately 97% effective in preventing measles. However, pockets of under-vaccination persist, especially in counties with high rates of personal belief exemptions or limited healthcare access. These gaps in coverage compromise herd immunity and create opportunities for outbreaks to occur and spread (Kuppalli and Perl 2025; Dyer 2025; Schweitzer 2025).

Beyond vaccination rates, other demographic and environmental variables have been linked to the spatial distribution of measles. Population density, for instance, influences the rate of person-to-person contact and can amplify transmission in urban areas. Conversely, rural counties may experience challenges related to surveillance, diagnosis, and healthcare delivery. Environmental factors such as temperature and precipitation may also affect transmission dynamics indirectly, by influencing human behavior or the stability of the virus in different conditions. Incorporating these diverse influences into spatial models allows for a more accurate assessment of risk and enhances the ability to predict future outbreaks (Bharti et al. 2011; Metcalf et al. 2017; Dey et al. 2024; Bidari and Yang 2024; Jia et al. 2023).

To address these challenges, advanced spatial models, e.g., hierarchical spatial models with Bayesian inference, have become increasingly important tools in public health research. By modeling spatial dependence and integrating covariates, these approaches provide smoothed estimates of disease incidence and support identification of high-risk areas, with many extensions developed in spatio-temporal settings (Rushworth et al. 2016; Sahu 2021; Chou-Chen et al. 2023; Retegui et al. 2024). However, modeling disease outbreaks frequently involves addressing sparse data, where many areas report zero cases while a few exhibit sudden surges. This pattern is particularly characteristic of measles, a disease largely contained through widespread vaccination but still prone to outbreaks in under-immunized populations. The Zero-Inflated Poisson (ZIP) model is particularly well-suited to handle such data structures, as it accommodates the overabundance of zeros-representing areas without reported cases-while simultaneously modeling count data where outbreaks do occur (Musal and Aktekin 2013; Osei et al. 2022; Arima et al. 2025). In the context of measles in Texas, ZIP models can provide a powerful tool for identifying regions with elevated outbreak potential, thereby informing targeted public health interventions such as enhanced surveillance, vaccination efforts, and community education initiatives. Therefore, in this study, we investigate the spatial distribution of measles outbreaks in Texas by utilizing a ZIP model enhanced with spatial dependence through a Conditional Autoregressive (CAR) prior. We consider two commonly used CAR priors, Besag–York–Mollié (BYM) model (Besag et al. 1991), and the Leroux CAR model (Leroux et al. 1999). However, we focus on purely spatial modeling, as the available data do not support a more detailed spatio-temporal analysis.

The model integrates key demographic variables, including MMR vaccination rates and population density, along with environmental factors such as temperature and precipitation. By applying the fitted ZIP model with CAR structure, we evaluate the spatial burden of measles using essential epidemiological indicators, including the standardized incidence ratio, relative risk estimates, and exceedance probabilities. These metrics provide a comprehensive framework for understanding geographic variations in measles incidence, capturing the influence of demographic and environmental heterogeneity across Texas counties.

The remainder of this paper is organized as follows. Section 2 describes the proposed methodology for modeling measles outbreak patterns in Texas, USA. Section 3 presents the data, details the modeling results, and quantifies the associated measles risks. Finally, Sect. 4 provides concluding remarks and discusses the broader implications of the findings.

## Methods

### Zero-inflated Poisson model (ZIP) with spatial dependence

Let $Y_i \in \mathbb {N}$ be the observed count of measles for Texas county *i*, where i=1,2,…,n. Here, $Y_i$s are observed with an excessive number of zeros. Therefore, we model measles count data with the zero-inflated Poisson model (ZIP) (Lambert 1992). The ZIP model combines two generating processes. The first process generates zeros with probability $p_i$, and the second process is governed by a Poisson distribution with probability $1-p_i$ that generates counts, some of which may be zeros. That is, we assume the observed measles counts $Y_i$ follow a mixture of two processes as

$$\begin{aligned} Y_i \sim {\left\{ \begin{array}{ll} 0 & \text {with probability } p_i \\ \text {Poisson}(\lambda _i) & \text {with probability } 1 - p_i \end{array}\right. } \end{aligned},$$

where $\lambda _i >0$ is the expected Poisson count for the county *i*, and $0 \le p_i \le 1$. We can write the probability mass function (pmf) of $Y_i$ as

1 $$\begin{aligned} P(Y_i = y_i | p_i, \lambda _i) = {\left\{ \begin{array}{ll} p_i + (1 - p_i) e^{-\lambda _i} & \text {if } y_i = 0 \\ (1 - p_i) \frac{\lambda _i^{y_i} e^{-\lambda _i}}{y_i!} & \text {if } y_i\in \mathbb {N}^{+} \end{array}\right. } \end{aligned},$$

where $y_i$ is a realization of $Y_i$. The expected value of $Y_i$ is $E[Y_i] = (1 - p_i) \lambda _i$, where $p_i$ is the probability of an excess zero, and $\lambda _i$ is the mean of the Poisson component. This result follows from the fact that $Y_i$ is zero with probability $p_i$, and follows a Poisson distribution with mean $\lambda _i$, otherwise. The variance of $Y_i$ can be written as $\text {Var}(Y_i) = \lambda _i(1 - p_i) (1 + p_i \lambda _i)$. The variance accounts for the overdispersion introduced by the zero-inflation component. The presence of $p_i$ increases variability compared to a standard Poisson model, making ZIP models useful for data with more zeros than expected under a Poisson assumption (Musal and Aktekin 2013; Young et al. 2022).

We can define the Poisson mean $\lambda _i$ as a function of covariates and spatial random effect as

2 $$\begin{aligned} \log (\lambda _i) = \log (E_i) + \beta _0+ \sum _{j=1}^{m}\beta _j X_{ji} + \psi _i \end{aligned},$$

where $E_i$ is the expected measles counts in county *i*, which is computed as the total measles counts in Texas multiplied by the relative population size in county *i*. Here, $X_1, X_2, \ldots , X_m$ are *m* covariates, $\beta _1, \beta _2, \ldots , \beta _m$ are regression coefficients, $\beta _0$ is an intercept term. The $\psi _i$ represents a spatial structure component for county *i*, which incorporates any spatial autocorrelation that remains in the data after the covariate effects have been accounted for.

The spatial structure component $\mathbf {\psi }=(\psi _1, \psi _1, \ldots , \psi _n)$ is evaluated using a Conditional Autoregressive (CAR) model via an n×n adjacency matrix *W*. The elements of *W* are defined as

3 $$\begin{aligned} w_{ik} = {\left\{ \begin{array}{ll} 1 & \text {if counties } i \text { and } k \text { share a common border } \\ 0 & \text {otherwise} \end{array}\right. } \end{aligned},$$

where i,k=1,2,…,n, $w_{ii} = 0$ for all *i*. In this paper, we consider two CAR models, i.e., the Leroux CAR model (Leroux et al. 1999), and the Besag–York–Mollié (BYM) CAR model (Besag et al. 1991).

- **Leroux CAR Model.** Based on the adjacency matrix *W* and positive spatial correlation ρ, $0 \le \rho \le 1$, the spatial dependencies can be evaluated using the CAR distribution as
  4 $$\begin{aligned} \psi _i \mid \mathbf {\psi }_{-i}, \rho , \tau ^2, W \sim N \left( \frac{\rho \sum _{k=1}^{n} w_{ik} \psi _k}{\rho \sum _{k=1}^{n} w_{ik} + (1-\rho )}, \frac{\tau ^2}{\rho \sum _{k=1}^{n} w_{ik} + (1-\rho )} \right) \end{aligned},$$
  where $$\mathbf {\psi }_{-i} = (\psi _1, \ldots , \psi _{i-1}, \psi _{i+1},\ldots , \psi _n)$$, $$\tau ^2$$ is the variance of the spatial random effect. The conditional expectation of $$\psi _i$$ is a weighted average of adjacent $$\psi _k$$. Expression (4) is called the Leroux CAR Model (Leroux et al. 1999). The resulting joint distribution of the spatial random effects $$\boldsymbol{\psi } = (\psi _1, ..., \psi _n)$$ can be written as $$\boldsymbol{\psi } \sim N(0, \tau ^2 Q(\rho , W)^{-1}),$$ where $$Q(\rho , W) = \rho (D - W) + (1 - \rho ) I$$, D is a diagonal matrix, i.e., $$D_{ii} = \sum _k w_{ik}~\text {(the number of neighbors of county { i})}$$, and I is the identity matrix. A key advantage of the Leroux CAR Model is that it avoids singularities in computation, which contributes to improved convergence in MCMC and Bayesian models. For spatial correlation ρ=1, the model corresponds to the Intrinsic CAR Model (ICAR) (Besag 1974), and ρ=0 corresponds to no spatial correlation (Banerjee et al. 2014; Rushworth et al. 2016; Sahu 2021).
- **BYM CAR model.** The BYM CAR model includes both structured spatial effect and unstructured random effect as $\psi _i = \phi _i + \theta _i,$ where $\theta _i$ is the unstructured spatial effect, i.e., i.i.d. error, which follows a normal distribution with zero mean and constant variance $\sigma ^2$, $\theta _i \sim \mathcal {N}(0, \sigma ^2)$, i=1,2,…,n. The structured spatial effect $\phi _i$ is modeled using the CAR distribution as
  5 $$\begin{aligned} \phi _i \mid \phi _{-i}, W, \tau ^2 \sim \mathcal {N}\left( \frac{\sum _{i=1}^n w_{ik} \phi _i}{\sum _{i=1}^n w_{ik}}, \frac{\tau ^2}{\sum _{i=1}^n w_{ik}} \right) . \end{aligned}$$
  Expression (5) is an Intrinsic CAR Model (ICAR) model with spatial correlation ρ=1.

We assign log-Gamma priors to the precision parameters, a normal prior to logit(ρ) (note that in the BYM CAR model, ρ is fixed at ρ=1), and independent normal priors to each regression parameter $\beta _j$ (j=1,2,…,m) and the intercept $\beta _0$ as

$$1/\sigma ^2 \sim \text {Inv-Gamma}(a_1, b_1) , \quad 1/\tau ^2 \sim \text {Inv-Gamma}(a_2, b_2),$$

$$\text {logit}(\rho ) = log \left( \frac{\rho }{1-\rho }\right) \sim \mathcal {N}(\mu _{\rho }, \nu _{\rho }), \quad \beta _0 \sim \mathcal {N}(\mu _0, \nu _0), \quad \beta _j \sim \mathcal {N}(\mu _j, \nu _j).$$

We model the zero-inflation probability $p_i$ using nonlinear logistic function as

6 $$\begin{aligned} \ln \left( \frac{p_i}{1-p_i}\right) = \gamma _0 + \sum _{j=1}^{m}\gamma _j Z_{ji}. \end{aligned} ,$$

where $Z_1, Z_2, \ldots , Z_m$ are *m* covariates that influence the probability of zero counts, $\gamma _1, \gamma _2, \ldots , \gamma _m$ are the corresponding coefficients, and $\gamma _0$ is an intercept. Similar to priors for $\beta _j$, we independently assign priors for each coefficient $\gamma _j$ as $\gamma _j \sim \mathcal {N}(\mu _j, \nu _j)$, and $\gamma _0 \sim \mathcal {N}(\mu _0, \nu _0)$. We can write the likelihood function of the associated parameters of the ZIP model as

$$\begin{aligned} L({\lambda }, {p}| {Y} ) = \prod _{i=1}^{n} \left[ p_i + (1 - p_i) e^{-\lambda _i} \right] ^{\textbf{1}(Y_i = 0)} \left[ (1 - p_i) \frac{\lambda _i^{Y_i} e^{-\lambda _i}}{Y_i!} \right] ^{\textbf{1} (y_i\in N^{+})}, \end{aligned}$$

where $\textbf{1}(\cdot )$ is the indicator function, $\lambda _i$ is given by  (2). We combine the likelihood function with the prior distributions to obtain the posterior distributions of model parameters. We perform the Bayesian estimation procedure based on the integrated nested Laplace approximation (INLA) (Rue et al. 2009, 2017) using the R package *R-INLA* (Bivand et al. 2015).

### Disease risk

We estimate the measles disease risk and compare its occurrences across different counties using *Standardized Incidence Ratios* (SIR), *relative risks* (RRs), and *Exceedance probabilities*. We can define these disease risk metrics as follows.

- **Standardized Incidence Ratios (SIR).** The Standardized Incidence Ratio (SIR) compares the observed number of measles cases in a given population to the number of expected cases based on a reference or standard population. SIR identifies the counties with statistically significant risk deviations from the expected disease burden. If $Y_i$ and $E_i$ are the observed and expected numbers of measles cases, respectively, in county *i*, i=1,2,…,n, the $SIR_i$ is defined as the following ratio:
  $$\ SIR_i = \frac{Y_i}{E_i}.$$
  For a given county i, an $SIR_i$ of 1.0 indicates that measles incidence in the county *i* aligns with that of the reference population. An $SIR_i > 1$ means the observed cases exceed the expected cases, identifying county i as a high-risk area. Conversely, an $SIR_i < 1$ indicates fewer observed cases than expected, suggesting county i is a low-risk area (Lawson 2014).
- **Relative Risks (RRs).** The relative risk is defined to quantify the relative incidence of measles disease for county *i*, i=1,2,…,n, as
  $$\ RR_i = \frac{(1 - \hat{p}_i) \hat{\lambda }_i}{E_i},$$
  where the numerator represents the estimated number of measles cases in county *i* based on the fitted ZIP model with spatial dependence, and $E_i$ is the expected number of measles cases.
- **Exceedance probabilities.** The exceedance probability for county *i* estimates the likelihood that its relative risk of measles counts exceeds a predefined threshold *c*. By setting *c*, we quantify the probability that the true disease risk in a county is above this level, thereby identifying high-risk areas with statistical confidence. In this study, we set c=1, so the exceedance probability is P(RR>1), representing the probability that the relative risk in a county exceeds the state average (Lawson 2018; Lee 2020; Moraga 2023).

## Results

In this section, we describe the data and evaluate the relevance of various covariates for modeling measles spread in Texas. We then conduct extensive experiments to identify the most suitable spatial model for quantifying measles outbreaks. Finally, we apply the selected model to assess measles risk across the state.

### Data

County-level measles case data in Texas, covering the period from late January 2025 to March 28, 2025, are obtained from the Texas Department of State Health Services (DSHS 2025). This source also provides data on county-level MMR vaccination rates. Climate data, including temperature and precipitation, are collected from the National Centers for Environmental Information (NCEI) (National Centers for Environmental Information (NCEI) 2025). The county-level population data are obtained from the United States Census Bureau (Census Bureau 2024), and the Texas county boundary shapefile, which aids in spatial analysis, is published by the Texas Department of Transportation (TxDOT) (Texas Department of Transportation (TxDOT) 2025).

We hypothesize that the spread of measles disease is influenced by county-level MMR vaccination rates, population size, age, and environmental factors, such as average temperature and precipitation. We define the focus variables in Table 1.

**Table 1:** Variable definitions

Variable	Description
Measles count (*Y*)	Number of reported measles cases per county
MMR Vaccination Rate ($X_{1}$)	Percentage of the population vaccinated against measles
Average Temperature ($X_{2}$)	Mean temperature during the study period
Average Precipitation ($X_{3}$)	Mean precipitation during the study period
$log_{10}$(Population Size) ($X_{4}$)	Total population of each county on the $log_{10}$ scale
Age ($X_{5}$)	Age category of each reported measles case

### Descriptive analysis

**Fig. 1:**
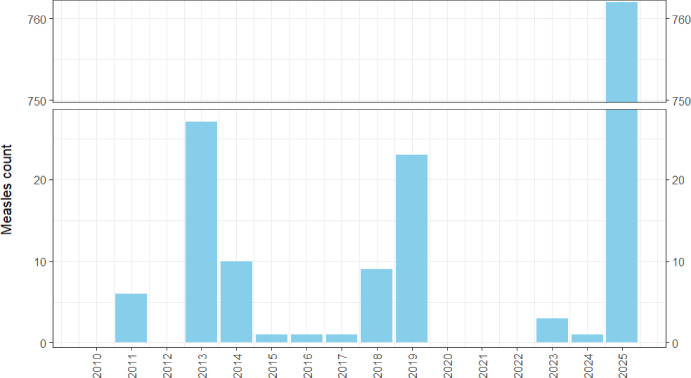
Total measles incidents in Texas between 2010 and 2025

Before analyzing the 2025 measles outbreak, we first examined temporal patterns in reported cases. Figure 1 displays annual measles counts in Texas from 2010 to 2025. We observe substantial temporal heterogeneity, with a pronounced spike in 2025, when reported cases exceeded 750, the highest level observed during the study period. In contrast, several years, including 2015–2017 and 2020–2024, recorded few or no cases. In Appendix A (Fig. 8), we present county-level maps for 2023 and 2024, which illustrate a spatially sparse incidence pattern, with most counties reporting zero cases. We also find that the set of counties with nonzero counts varies across years. In light of this temporal and spatial pattern, we focus the remainder of the analysis on the 2025 outbreak data.

**Fig. 2:**
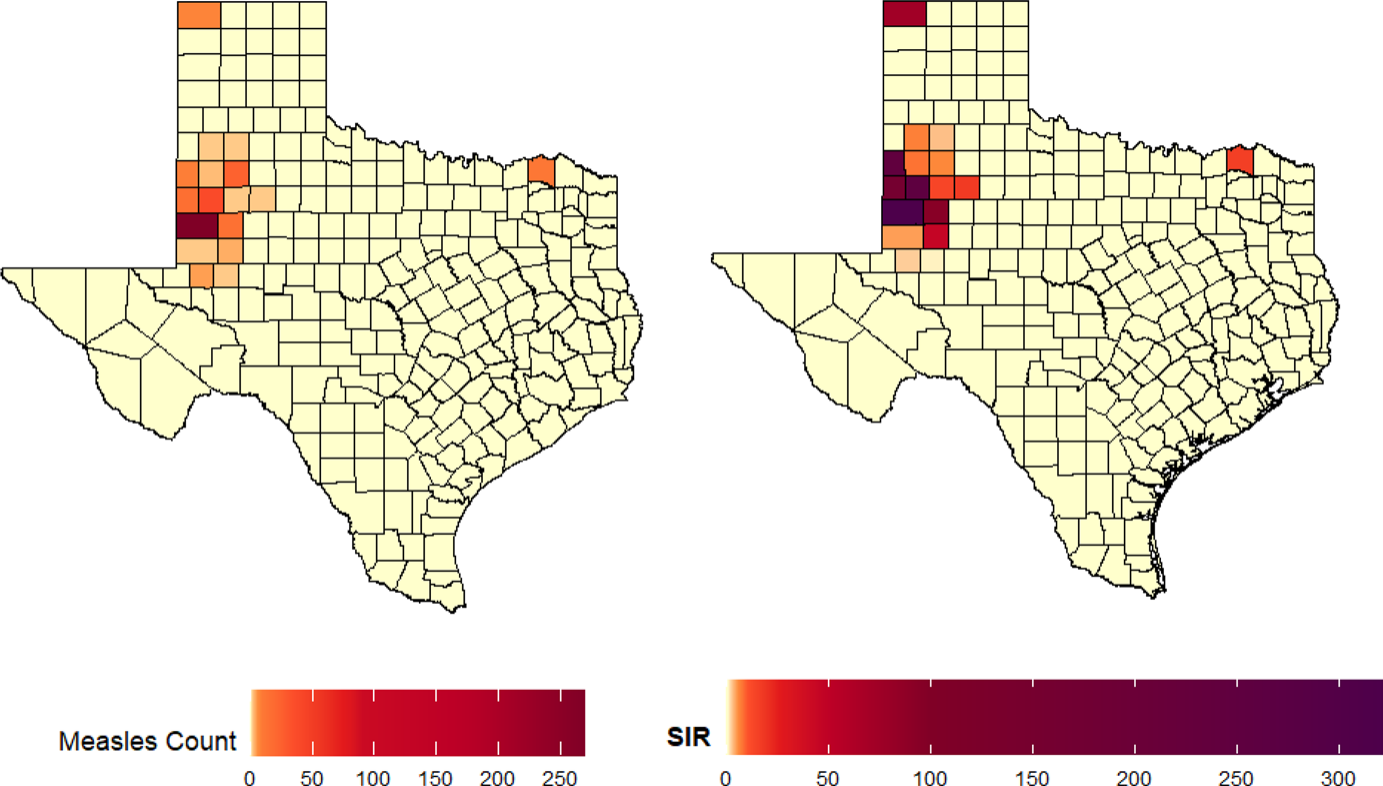
Spatial patterns of the measles counts and SIR values

**Fig. 3:**
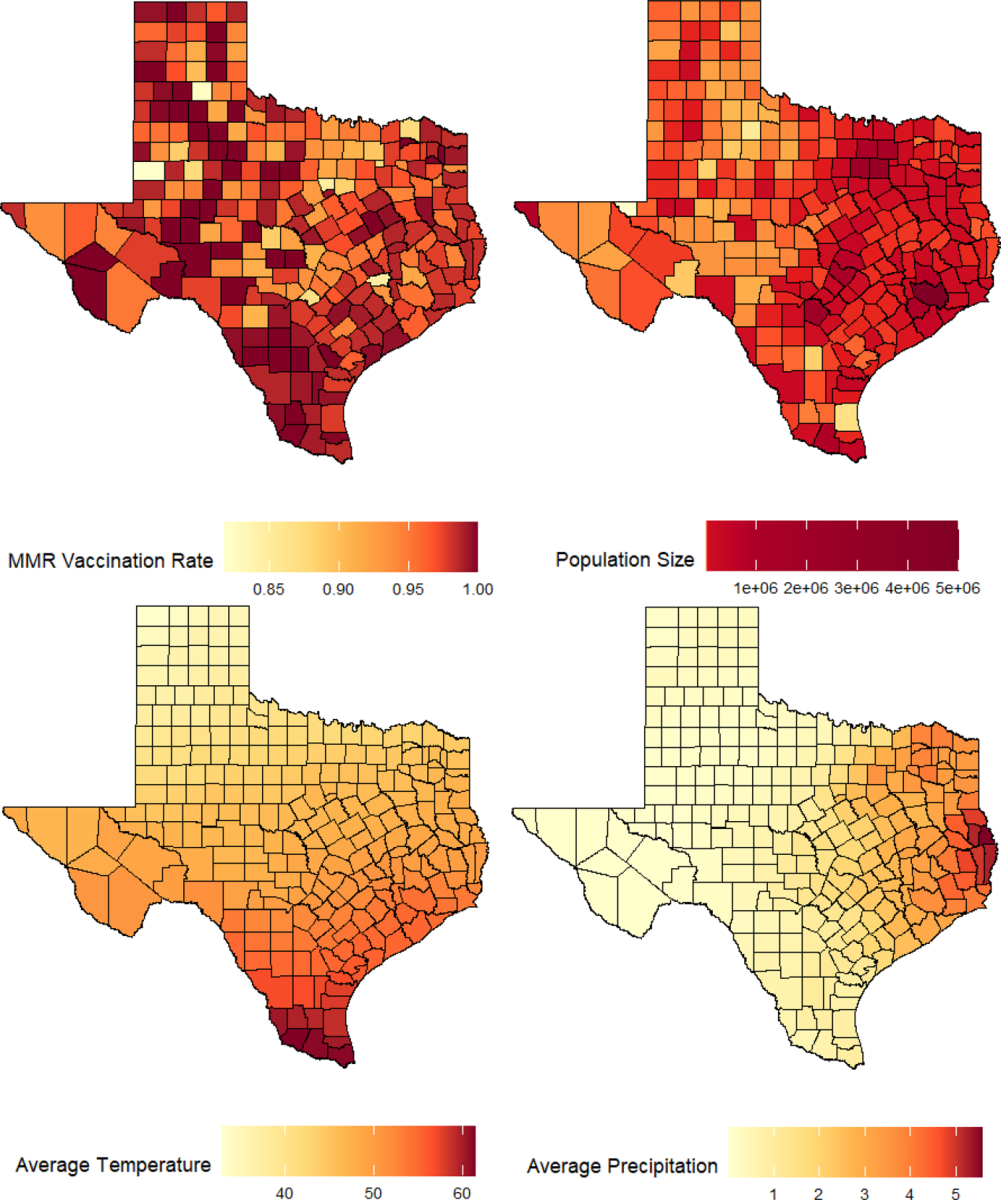
Spatial distribution of demographic, epidemiological, and climate variables

Figures 2 and 3 illustrate the spatial distribution of key variables across all 254 counties in Texas in 2025. The maps depict the measles disease count, SIR values, MMR vaccination rate, population size, average temperature, and average precipitation, providing a comprehensive overview of the geographic patterns of these factors. The color gradients in the maps represent the distribution of the respective variables, where lighter shades indicate the lower values, and darker shades correspond to the higher values of the variables, highlighting regional variations in measles incidence, SIR values, MMR vaccination rates, population size, average temperature, and average precipitation.

The left panel of Fig. 2 illustrates the spatial distribution of measles cases across Texas counties, while the right panel highlights the concentration of elevated SIR values predominantly within the northwestern region of the state. These counties exhibit a significantly higher incidence of measles compared to the state average, marking them as key hotspots of disease activity and increased vulnerability. Additionally, Fig. 2 demonstrates that areas with a high incidence of measles cases, particularly in the northwestern region, typically correspond with a moderate MMR vaccination rate (see the upper left panel of Fig. 3). This suggests that while vaccination coverage is not critically low, it may still be insufficient to achieve herd immunity, leaving pockets of the population vulnerable to outbreaks.

**Fig. 4:**
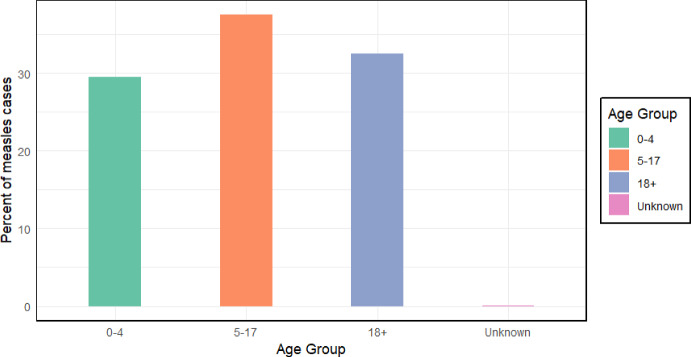
Percentage of measles cases in Texas by age for the year 2025

We next evaluate the age-specific distribution of measles cases. Figure 4 presents the percentage of reported cases across the state of Texas in 2025 by age group and reveals a clear concentration among children. Despite spanning the narrowest age interval, children aged 0–4 years account for roughly 30% of all cases, indicating a high intensity of infection in early childhood. The 5–17 year age group, corresponding to school-aged children, represents the largest share, at approximately the mid- to high-30% range. This pattern is consistent with elevated contact rates in school settings and suggests sustained transmission within these environments. Adults aged 18 years and older comprise a somewhat smaller, though still substantial, proportion of cases, while cases with unknown age are negligible. A chi-square test showed that measles cases in Texas during 2025 were not evenly distributed across age groups ($\chi ^2$ = 254.68, df = 3, *p*-value <0.01). Overall, the figure indicates that measles incidence in 2025 is strongly concentrated among children, with particularly high burdens in early childhood and the school-aged population.

**Fig. 5:**
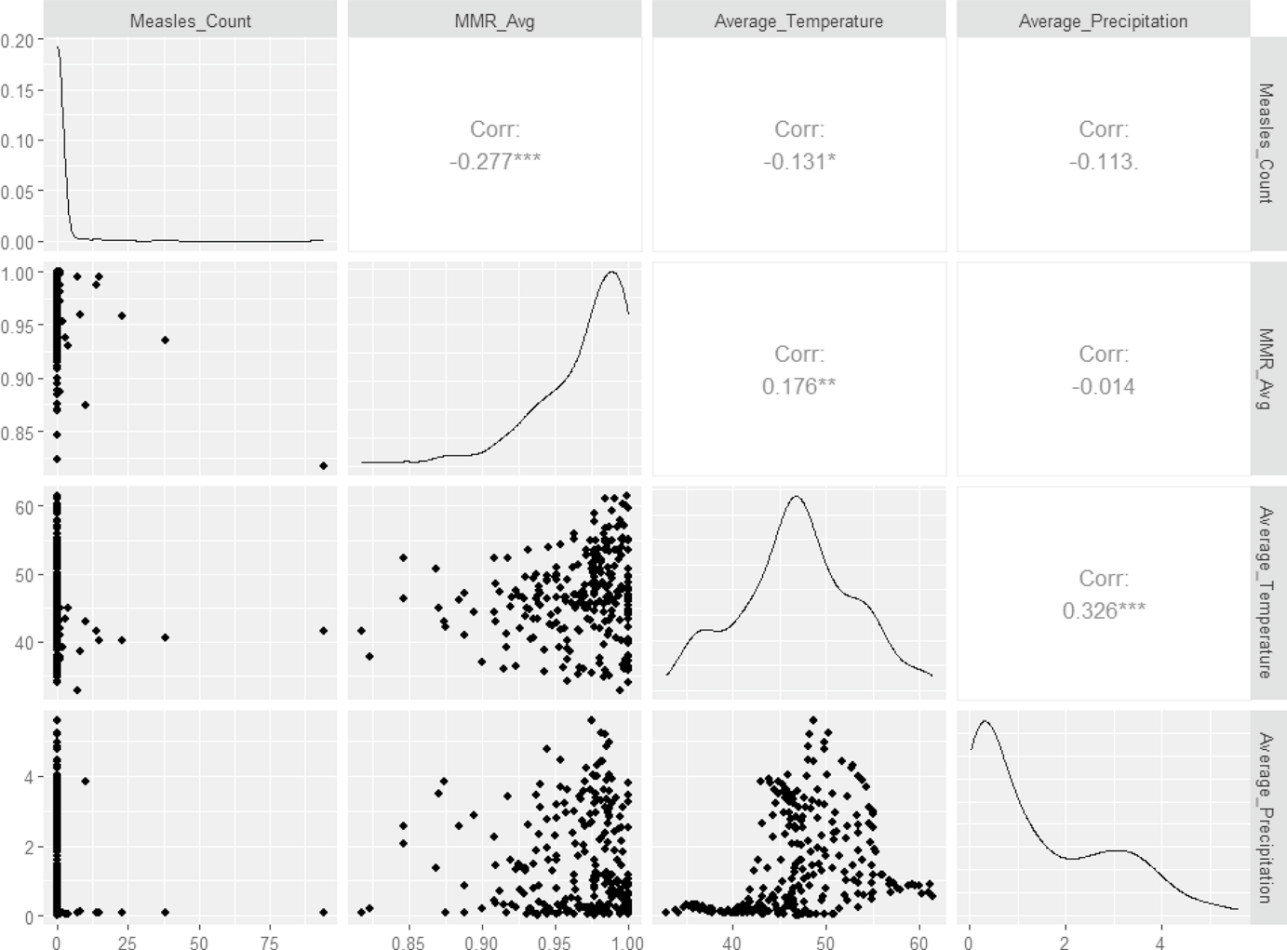
Scatterplot showing the relationships in the data in 2025

Figure 5 shows scatter plots, density distributions, and correlation coefficients to illustrate the relationships between variables. We find that the correlations between measles count and the explanatory variables are all negative and statistically significant. The level of spatial autocorrelation in these variables is assessed using *Moran’s I statistic*, which quantifies the degree to which a variable is clustered, dispersed, or randomly distributed across its neighbors. The Moran’s *I* statistic ranges from − 1 to 1, where values close to 1 suggest strong spatial clustering (similar values are near each other), values near -1 indicate spatial dispersion (dissimilar values are near each other), and values around 0 imply a random spatial pattern (Moran 1950; Cliff and Ord 1973; Li 2023). The corresponding Moran’s *I* statistics and *p*-values for all variables are summarized in Table 2. We find strong evidence of statistically significant positive spatial autocorrelation in measles counts across Texas counties, as well as significant spatial autocorrelation in MMR vaccination coverage. Mean temperature and mean precipitation also exhibit strong spatial autocorrelation, indicating that nearby counties have very similar temperature and precipitation patterns.

**Table 2:** Moran’s *I* values

Variable	Moran’s *I* value	*p*-value
Measles disease count	0.299	<0.01
MMR vaccination rate	0.103	<0.01
Average temperature	0.975	<0.01
Average precipitation	0.971	<0.01

### Model selection

We have conducted extensive experiments to identify the best-performing Bayesian spatial model for quantifying measles outbreaks in Texas, USA. We update the Poisson mean $\lambda _i$  of (2) as a function of covariates, county-level MMR vaccination rate $X_{1}$, mean temperature $X_{2}$, mean precipitation $X_{3}$, and spatial random effect as

7 $$\begin{aligned} \log (\lambda _i)=\log (E_i) + \beta _0 + \beta _1 X_{1i} + \beta _2 X_{2i} + \beta _3 X_{3i} + \phi _i. \end{aligned}$$

For the age variable ($X_{5}$), we only have statewide aggregated data, as shown in Fig. 4 of Sect. 3.2. County-level age-specific measles counts are not available; consequently, we are unable to include age as a covariate in the county-level spatial model.

For a comprehensive evaluation, we focus on the following three spatial models that demonstrate the strongest performance.

- Model 1: ZIP model without spatial dependence (ZIP + IID)
- Model 2: ZIP model with BYM spatial dependence (ZIP + ϕ (BYM))
- Model 3: ZIP model with Leroux spatial dependence (ZIP + ϕ (Leroux))

All three models, with or without spatial dependence (ϕ), include the three covariates specified in  (7). In the models, the zero-inflation probability $p_i$ (i.e., ZIP models) is evaluated using  (6). However, since we mainly focus on covariates’ effect on the Poisson mean, we model $p_i$ using an intercept term as $\ln \left( \frac{p_i}{1-p_i}\right) =\gamma _0$.

Based on the information provided in Bivand et al. (2015), we specify the priors of the candidate models based on the following distributions:

$$1/\sigma ^2 \sim \text {{Inv-Gamma}(1, 0.0005)} , \quad 1/\tau ^2 \sim \text {{Inv-Gamma}(1, 0.0005)},$$

$$\text {logit}(\rho ) = log \left( \frac{\rho }{1-\rho } \right) \sim \mathcal {N}(0,0.1),$$

$$\beta _0 \sim \mathcal {N}(0, 1000), \quad \beta _j \sim \mathcal {N}(0, 1000), \quad \gamma _0 \sim \mathcal {N}(0, 1000).$$

We conduct the model comparisons based on the commonly used *Watanabe–Akaike information criterion* (WAIC). The WAIC is a Bayesian model selection metric that improves upon the deviance information criterion (DIC) (Akaike 1974; Spiegelhalter et al. 2002) by fully integrating over the entire posterior distribution, making it more robust for complex models with non-Gaussian posteriors (Watanabe 2010, 2013; Meyer 2016). A lower WAIC indicates a better trade-off between fit and complexity.

Table 3 presents the performance metrics of the competing models, illustrating their ability to capture county-level measles outbreaks in Texas. We find that the ZIP model with BYM spatial dependence (Model 2) outperforms all other models, yielding the lowest WAIC value. In contrast, the Leroux model yields a substantially larger WAIC. Under the highly sparse, zero-dominated county-level data, the Leroux specification exhibits numerical instability and weak identification of the spatial dependence parameter, which likely contributes to its poorer WAIC performance.

**Table 3:** Model comparison according to WAIC

Model number	Model	WAIC
Model 1	ZIP + IID	167.54
Model 2	**ZIP +** ϕ (BYM)	**156**.**58**
Model 3	ZIP + ϕ (Leroux)	28747.54

Bold fonts imply that the corresponding models are the best

We perform Bayesian estimation utilizing the INLA approach through the R package *R-INLA* (Bivand et al. 2015). The computation time required for this analysis is approximately 2.5 s on a workstation that is equipped with 16 GB of RAM and a 1.70 GHz Intel Core i7 processor.

### Simulation Study

In this section, we conduct an experiment to assess our models for simulated data. For each county i, i=1,2,…,254, we generate three covariates, $X_1$, $X_2$, and $X_3$, from normal distributions as

$$X_{1i} \sim \mathcal {N}(\mu _{\text {vacc}}, 0.05), \quad X_{2i} \sim \mathcal {N}(\mu _{\text {temp}}, 0.05), \quad X_{3i} \sim \mathcal {N}(\mu _{\text {precp}}, 0.05),$$

where $\mu _{\text {vacc}}$, $\mu _{\text {temp}}$, and $\mu _{\text {precp}}$ represent the observed vaccination rate, average temperature, and average precipitation for county i, respectively. We also simulate the number of measles cases in each county using a ZIP model as $Y_i \sim \mathcal {ZIP}(\pi , y_{0i}),$ where $\pi = {\exp (\gamma _0)}/{1 + \exp (\gamma _0)}$, $\gamma _0 = 0.730$ is taken from the fitted Model 2, and $y_{0i}$ are the observed measles counts for county i. This setup ensures that the simulated data reflect both the observed covariate structure and the ZIP model parameters estimated from the real data.

Table 4 reports the mean WAIC values and their standard deviations based on 2,000 simulations. Consistent with the results from the observed data, Model 2 demonstrates the best performance.

**Table 4:** Model comparison using simulated data according to WAIC with ϕ based on either BYM or Leroux

Model number	Model	WAIC
Model 1	ZIP + IID	15648.95 (83013.68)
Model 2	**ZIP +** ϕ (BYM)	**12471**.**82** (71739.71)
Model 3	ZIP + ϕ (Leroux)	56760.47 (275847.1)

Bold fonts imply that the corresponding models are the best

Standard errors are in the parentheses

### Measles risk

Table 5 presents the posterior mean estimates of the parameters of the selected ZIP model with BYM spatial dependence (Model 2) along with their 95% credible intervals. A parameter is considered statistically significant if its 95% credible interval does not include zero. We find that all the parameters are statistically significant. MMR vaccination rate, temperature, and precipitation are found to have negative effects on measles outbreaks across counties.

We interpret the estimated regression coefficient $\beta _j$ in terms of the relative risk, given by $\exp (\beta _j)$, for j=1,2,…,m. More generally, the relative risk for a *d*-unit increase in a covariate with regression parameter $\beta _j$ is $\exp (d \cdot \beta _j)$. The output in Table 5 shows that the posterior median and 95% credible interval for the relative risk of a 5% increase in MMR Vaccination rate is 0.204 (0.083, 0.492), suggesting a decrease of 80% in measles counts. That is, higher MMR vaccination rates directly contribute to herd immunity, reducing the likelihood of measles transmission. The corresponding relative risk for a 10-degree increase in temperature is 0.003, indicating that measles counts are 99.70% lower for such an increase. This analysis is based on January-March data, meaning that higher winter temperatures are strongly associated with reduced measles incidence, potentially reflecting temperature’s role in suppressing transmission during cooler months. In addition, a one-unit increase in precipitation is associated with a 63% reduction in measles counts, indicating that wetter winter conditions also limit transmission. Together, these results underscore the influence of environmental factors on measles dynamics and reinforce the importance of maintaining high vaccination coverage alongside awareness of climate-related risks.

We also find that the zero-inflation probability $p_i$ has an intercept $\gamma _0 = 0.73$ on the logit scale. This corresponds to an estimated baseline zero-inflation probability of $p_i = \frac{\exp (0.73)}{1 + \exp (0.73)} \approx 0.675,$ indicating that about 67.5% of observations are generated by the structural-zero process, while the remaining 32.5% come from the Poisson count process, which can produce both positive counts and random zeros. In other words, for a typical county–year at baseline conditions (no covariates), the model estimates a 67.5% chance of having no realistic possibility of measles cases.

**Table 5:** Parameter estimates with 95% credible intervals for Model 2

Variable	Parameter	Mean	95% credible interval
Intercept (for $\lambda _i$)	$\beta _0$	55.953	(35.041, 77.563)
MMR vaccination rate	$\beta _1$	− 0.318	(− 0.498, − 0.142)
Average temperature	$\beta _2$	− 0.573	(− 0.807, − 0.343)
Average precipitation	$\beta _3$	− 0.993	(− 1.876, − 0.139)
Intercept (for $p_i$)	$\gamma _0$	0.730	(0.634, 0.815)
Precision–random spatial effect	$1/\tau ^2$	2118.839	(676.034, 4581.227)
Precision–i.i.d. error	$1/\sigma ^2$	0.331	(0.204, 0.511)

Figure 9 in Appendix B displays the density plots of the precision of the i.i.d. error term $\theta _i$ (i.e., $1/\sigma ^2$) and the precision of the spatial random effect ($1/\tau ^2$) of the BYM spatial dependence. The trace plots of the coefficient estimates, shown in Fig. 10 in Appendix B indicate clear convergence of the Bayesian estimates. The smooth, unimodal density curves further support the convergence of the model parameters. In addition, Fig. 11 in Appendix B presents the autocorrelation function (ACF) plots for the posterior samples of the model parameters. Excluding lag 0, the ACFs are generally close to zero across lags, with a small number of correlations for some parameters slightly exceeding the approximate 95% bounds. This pattern indicates weak short-range dependence rather than evidence of poor mixing or convergence issues. We present county-level residuals from the selected ZIP + ϕ (BYM) model in Fig. 12 in Appendix B. Residuals are close to zero for the majority of counties, indicating strong agreement between observed and predicted measles incidence across the study region. A small number of counties exhibit larger negative residuals, suggesting modest overprediction in these areas, with no evidence of widespread or systematic lack of fit.

We have also studied the parameter estimates of Model 1, i.e., ZIP+IID model (Table 6 in Appendix C). We find that all covariates remain statistically significant under the ZIP+IID specification, with effect sizes that are very similar to those obtained from the ZIP + ϕ (BYM) model. This similarity suggests that incorporating a spatial structure does not materially alter the estimated covariate effects. We compute county-level residuals from the ZIP+IID model and evaluate residual spatial structure using the global Moran’s *I* statistic. The residuals exhibit significant spatial autocorrelation (Moran’s *I* =0.188, *p*-value <0.01), indicating that neighboring counties tend to have similar residual values rather than behaving independently. This finding suggests that a spatially structured model is required to adequately account for the remaining spatial dependence.

**Fig. 6:**
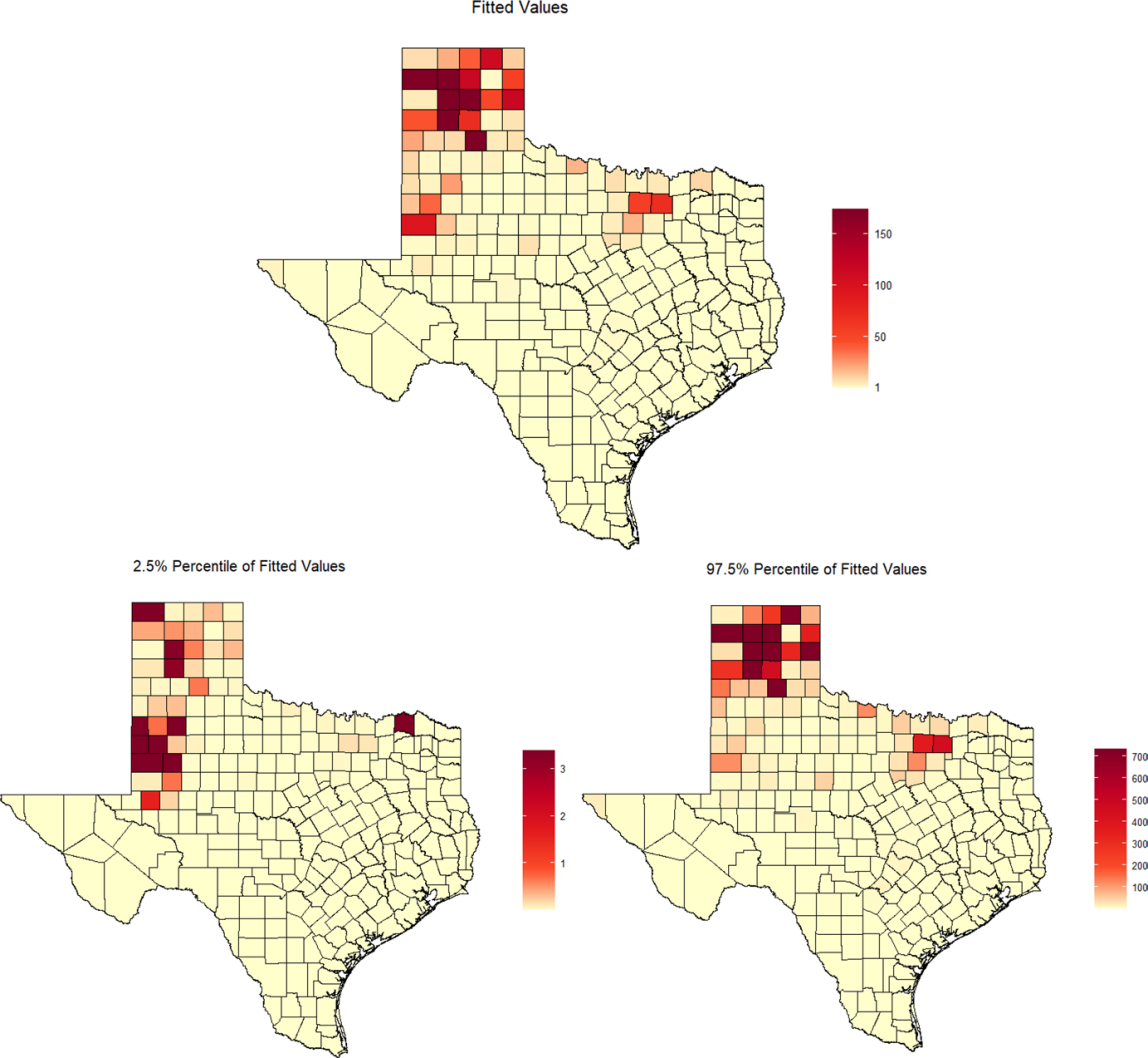
Predicted measles count (upper row) and 2.5 and 97.5% percentiles (bottom row) for each county, Texas

We proceed to quantify measles risk using the selected ZIP + ϕ (BYM) model. Figure 6 delivers a comprehensive spatial analysis of predicted measles counts across Texas counties, providing insights into both the central estimates and the associated uncertainty. The top map illustrates the fitted measles counts, indicating that northern Texas counties are poised to experience significantly higher incidence rates compared to other regions. The use of a color gradient, from light yellow to dark red, communicates the intensity of predicted cases, with darker shades representing higher counts. This visualization identifies geographic hotspots and highlights areas that demand urgent attention for public health planning. The bottom two maps enhance our understanding of variability in these predictions by displaying the 2.5% and 97.5% percentiles.

When compared to the observed counts in Fig. 2 (left), the results highlight the model’s effectiveness in capturing a smoothed and realistic distribution of measles cases across the study area. By incorporating spatial relationships between neighboring counties and accounting for key covariates such as MMR vaccination rates, population density, temperature, and precipitation, the model effectively adjusts for both spatial dependence and the demographic and environmental factors influencing measles transmission.

**Fig. 7:**
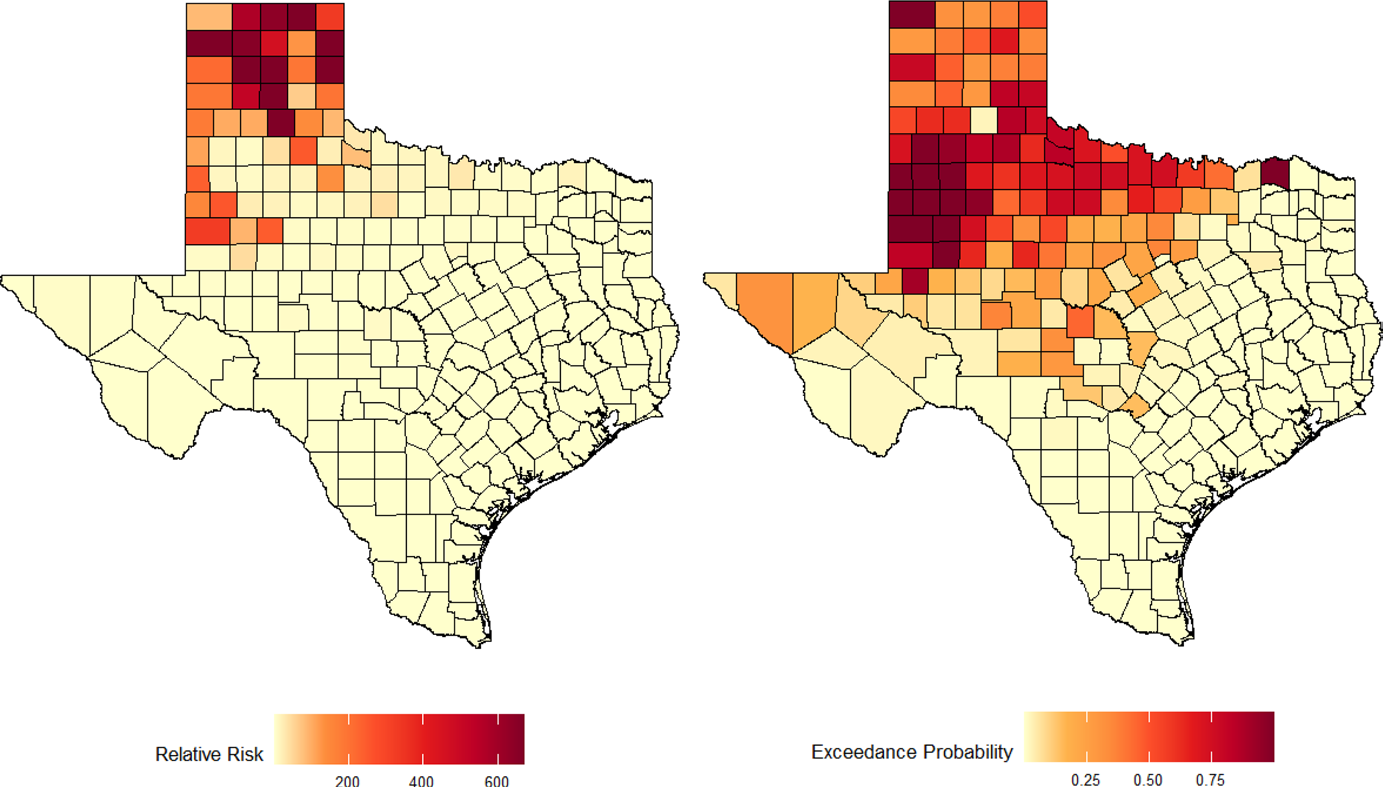
Mapping measles risks in Texas counties

**Fig. 8:**
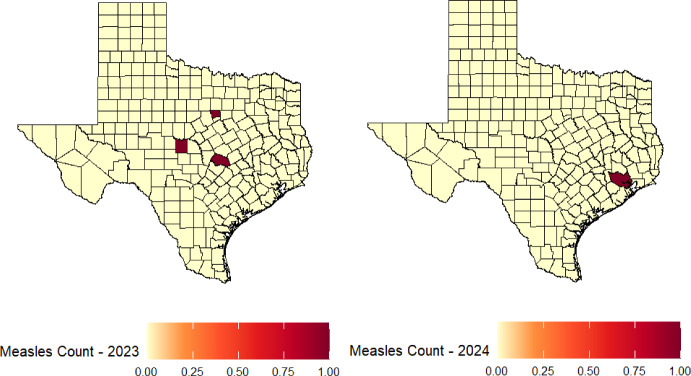
Spatial pattern of measles counts in Texas counties in 2023 and 2024

**Fig. 9:**
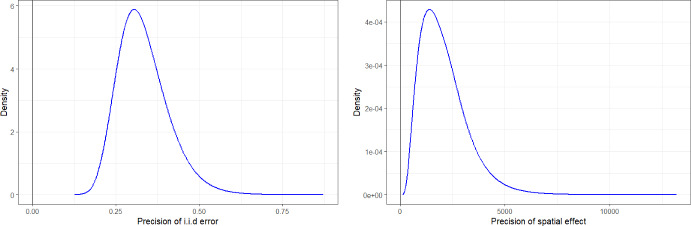
Density plot of the precision of i.i.d. error ($1/\sigma ^2$) (left panel), and precision of spatial random effect ($1/\tau ^2$)

**Fig. 10:**
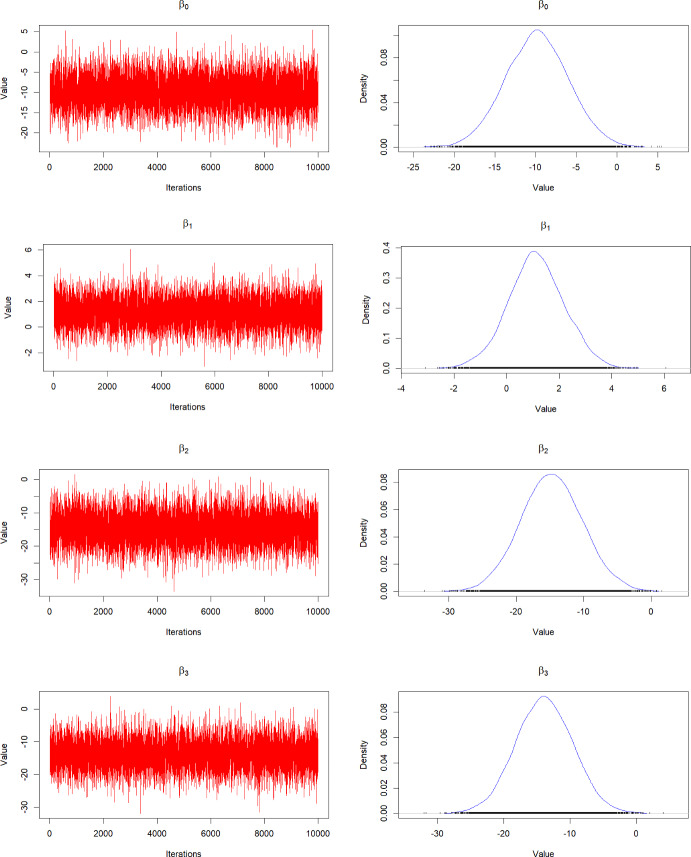
Diagnostic plots of the parameters for Model 2. The left panel contains trace-plots while the right panel contains density estimates

**Fig. 11:**
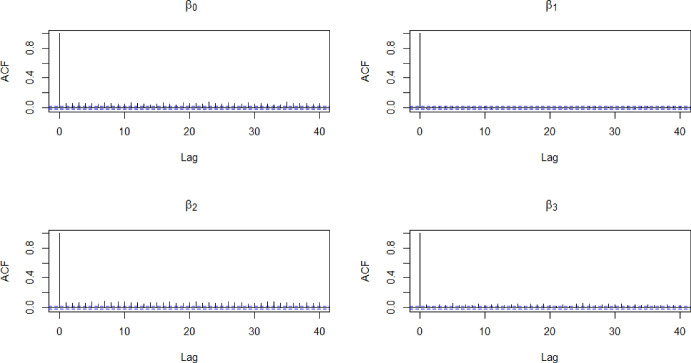
Autocorrelation functions (ACFs) for posterior samples of the parameters for Model 2

**Fig. 12:**
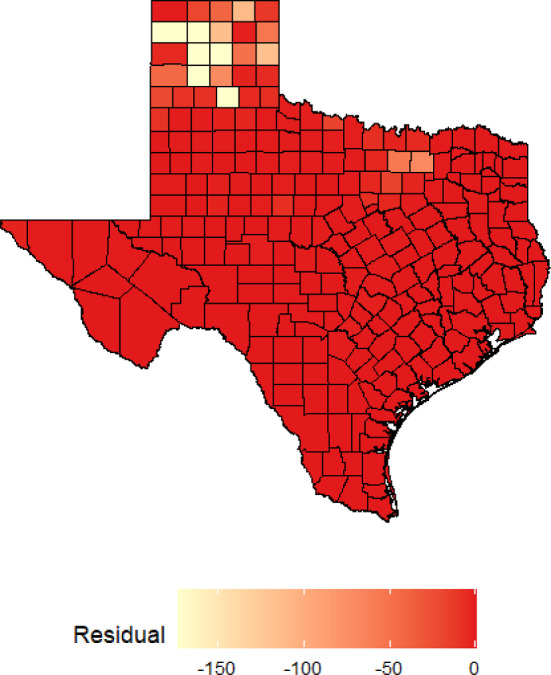
Residual maps for Model 2

Figure 7 illustrates the spatial patterns of the relative risk (RR) and the posterior exceedance probabilities of measles disease across Texas counties. The left panel displays the RR of measles, with a gradient from yellow to dark red indicating increasing levels of risk. Notably, the Panhandle region in northern Texas shows the highest RR, suggesting a greater likelihood of measles outbreaks in these counties compared to others. While both the RR and SIR metrics (shown in the right panel of Fig. 2) identify the Panhandle and northwestern regions as high-risk zones, the RR map reveals elevated risk across a broader range of counties, indicating a more widespread vulnerability. This broader prominence of RR may reflect underlying factors such as MMR vaccination rates, population density, temperature, and precipitation that are not fully accounted for by observed incidence alone.

The right panel of Fig. 7 presents the exceedance probability, defined as P(RR>1), which represents the likelihood that a county’s relative risk is higher than the state average. This metric offers a probabilistic perspective on the risk, employing a similar color gradient to indicate higher probabilities. The pattern closely mirrors that of the relative risk map, with northern Texas counties showing elevated exceedance probabilities.

## Conclusion

This study underscores the importance of hierarchical spatial modeling in understanding and responding to the resurgence of measles in Texas. Despite the long-standing availability of a highly effective vaccine, recent outbreaks highlight persistent vulnerabilities associated with under-vaccination, demographic heterogeneity, and environmental influences. Our modeling results provide clear empirical support for a spatially structured approach, as residuals from a non-spatial ZIP+IID model exhibit significant spatial autocorrelation, indicating that the assumption of independence across counties is untenable. Incorporating spatial dependence through a Bayesian Zero-Inflated Poisson model with a BYM CAR prior effectively accounts for this residual structure and yields improved model fit and predictive performance, as reflected by a lower WAIC. This framework accommodates the challenges posed by sparse count data while capturing latent spatial processes that influence measles transmission. The integration of key covariates, including MMR vaccination rates, population density, temperature, and precipitation, further refines our understanding of outbreak dynamics across Texas counties.

The results are consistent with previous measles risk modeling studies that document strong associations between outbreak risk and vaccination coverage, demographic structure, and environmental conditions (Yang et al. 2014; Hotez 2016; Ma et al. 2017). In particular, our spatial risk estimates identify elevated risk in regions with low MMR coverage, aligning with recent analyses of the 2025 Texas outbreak that report early transmission concentrated in under-vaccinated communities (González-Parra et al. 2025; Bi et al. 2025).

The findings of this study reinforce the critical role of both demographic and environmental factors in shaping transmission patterns and emphasize the utility of advanced spatial models in public health surveillance and planning. These insights can guide targeted vaccination campaigns, policy interventions, and resource allocation, ultimately strengthening measles prevention efforts and enhancing preparedness for future outbreaks.

Although this paper is grounded in county-level data from Texas, the modeling framework and substantive insights are broadly transferable to other regions facing similar measles resurgence. The hierarchical spatial structure is not specific to Texas, but rather reflects general features of infectious disease surveillance data, including excess zeros, spatial clustering, and unobserved contextual influences. In settings where vaccination coverage is uneven and populations are socially or geographically segmented, non-spatial models are likely to underestimate risk by ignoring spatial dependence. The observed improvement in fit after incorporating a BYM CAR prior suggests that latent spatial processes play an important role beyond measured covariates, a pattern that is expected to hold in other states or countries with decentralized public health systems and heterogeneous vaccine uptake.

Given the data limitations discussed in Sects. 3.2 and 3.3, the present analysis is necessarily purely spatial, focusing on the 2025 measles outbreak in Texas. As richer surveillance data become available, future work will extend this framework to spatio-temporal models.

## Data Availability

No datasets were generated or analysed during the current study.

## Appendix A: measles counts in Texas

Figure 8 illustrates county-level measles case counts in Texas for 2023 (left panel) and 2024 (right panel).

## Appendix B: model diagnostics: ZIP + ϕ (BYM)

Figure 9 displays density plots for the precision parameters of the i.i.d. error term and the spatial random effect in the fitted ZIP + ϕ (BYM) model (Model 2).

Diagnostic trace and posterior density plots for the model parameters are shown in Fig. 10, indicating satisfactory mixing and convergence. Autocorrelation functions (ACFs) for the fitted parameters are presented in Fig. 11. Figure 12 presents county-level residuals across all Texas counties.

## Appendix C: ZIP + IID Model

Table 6 reports the posterior mean estimates of the parameters for the ZIP+IID model (Model 1), together with their 95% credible intervals.

**Table 6:** Parameter estimates with 95% credible intervals for Model 1

Variable	Parameter	Mean	95% credible interval
Intercept (for $\lambda _i$)	$\beta _0$	55.652	(34.569, 77.840)
MMR vaccination rate	$\beta _1$	− 0.318	(− 0.506, − 0.141)
Average temperature	$\beta _2$	− 0.565	(− 0.802, − 0.336)
Average precipitation	$\beta _3$	− 1.030	(− 1.988, − 0.147)

## Code availability

All R codes are available upon request.
